# Quasi-Isostructural Co(II) and Ni(II) Complexes with Mefenamato Ligand: Synthesis, Characterization, and Biological Activity

**DOI:** 10.3390/molecules25133099

**Published:** 2020-07-07

**Authors:** Michał Gacki, Karolina Kafarska, Anna Pietrzak, Izabela Korona-Głowniak, Wojciech M. Wolf

**Affiliations:** 1 Institute of General and Ecological Chemistry, Faculty of Chemistry, Lodz University of Technology, 116 Zeromskiego Street, 90-924 Lodz, Poland; karolina.kafarska@p.lodz.pl (K.K.); anna.pietrzak.1@p.lodz.pl (A.P.); wmwolf@p.lodz.pl (W.M.W.); 2 Department of Pharmaceutical Microbiology, Medical University of Lublin, Chodzki 1, 20-093 Lublin, Poland; iza.glowniak@umlub.pl

**Keywords:** non-steroidal anti-inflammatory drug-complex, quasi-isotructural, thermal analysis, antioxidant activity, mefenamic acid

## Abstract

Three metal complexes of mefenamato ligand **1** were synthesized: [Co_2_(mef)_4_(EtOH)_2_(H_2_O)_4_]: **2**; [Co(mef)_2_(MeOH)_4_]∙2MeOH: **3**; and [Ni(mef)_2_(MeOH)_4_]∙2MeOH: **4**. Their compositions and properties were investigated by elemental analysis (EA), flame atomic absorption spectrometry (FAAS), Fourier-transform infrared spectroscopy (FTIR), and thermogravimetric analysis (TGA). Crystal structures were determined by the single crystal X-ray diffraction technique. Additionally, their antioxidant and antimicrobial activity were established, thus proving good/moderate bioactivity against Gram-positive bacteria and yeasts. In the crystal structure of **2**, an apical water molecule is shared between two adjacent cobalt(II) ions, resulting in the formation of a polymeric chain extending along the [100] direction. Meanwhile, structures **3** and **4** have strong intermolecular hydrogen bonds with diverse topologies that yield unique quasi-isostructural arrangements. The packing topology is reflected by the Hirshfeld surface analysis of intermolecular contacts.

## 1. Introduction

Nonsteroidal anti-inflammatory drugs (NSAIDs) are among the most popular pharmaceuticals that have been used against inflammation, pain, and fever over the years [1]. Their therapeutic effect is mainly based on their ability to inhibit the conversion of the arachidonic acid to prostaglandins [2]. Unfortunately, NSAIDs exhibit acute side effects and may cause dyspepsia, nausea, abdominal pain, constipation, headache, dizziness, and rash [2,3]. Additionally, several NSAIDs can be effectively applied in treatments of colon, lung, and breast cancers [4,5] due to their profound chemopreventive and chemosuppressive effects [6,7,8].

The structural similarity of crystals [9] plays a vital role in understanding the pharmaceutical activity of solid-state drugs. It is often described by the term isostructurality [10] and is profoundly associated with intermolecular interactions and often prompted by symmetry restrictions. A given packing arrangement may tolerate tiny molecular geometry adjustments while keeping the crystals almost isostructural [11,12]. However, certain crystals of coordination compounds which diverse metal ions are characterized by the same chemical constituents may not be classified as entirely isostructural due to subtle differences in their interaction arrangement [13]. That quasi-isostructurality was observed in systems **3** and **4**.

Mefenamic acid (2-(2,3-dimethylphenyl)aminobenzoic acid) **1** was discovered by Claude Winder from Parke–Davis in 1961 and successively introduced into the market as an NSAID three years later [14,15]. It belongs to the group of anthranilic acid derivatives also known as fenamates [16,17]. Nowadays, it is widely used as an analgesic and antipyretic agent characterized by mild side-effects and applied in treatments of osteoarthritis, rheumatoid arthritis, and other painful musculoskeletal illnesses [18,19].

Mefenamic acid adopts three distinctive polymorphic forms, denoted as I, II, and III [20,21]. They show diverse solubilities and stabilities [22,23]. Form I is the most stable under ambient conditions, II is stable above 160 °C, and III is the least stable and converts back to I [22]. The crystal structures of I, II, and III were reported in 1976, 2008, and 2012, respectively [22,23,24,25]. Mefenamic acid belongs to Class II of the Biopharmaceutical Classification System [26] and is characterized by a relatively low solubility and high permeability, both of which hamper its clinical use [22]. Its metal complexes may overcome this deficiency. In fact, considerable attention has been paid to transition metal complexes with active drugs [2,27]. Those studies showed that several properties, like solubility, reactivity, and stability, are different than those determined for the ligand or metal alone [28,29]. Metal complexes of fenamates, including those with mefenamic acid, have been recently investigated. Most of them have shown improved biological activities compared to the sole drugs [3,30,31] and revealed interesting antibacterial, antifungal, analgesic, and anti-inflammatory applications. 

Additionally, a number of crystal structures of metal–mefenamato complexes have been reported. In particular, a series of Mn(II) [3,32], Co(II) [33], Ni(II) [34], Cu(II) [35,36,37], Zn(II) [38], and Sn(IV) [39] complexes showed that mefenamato ligands may be involved in diverse coordination modes.

**Scheme 1:**
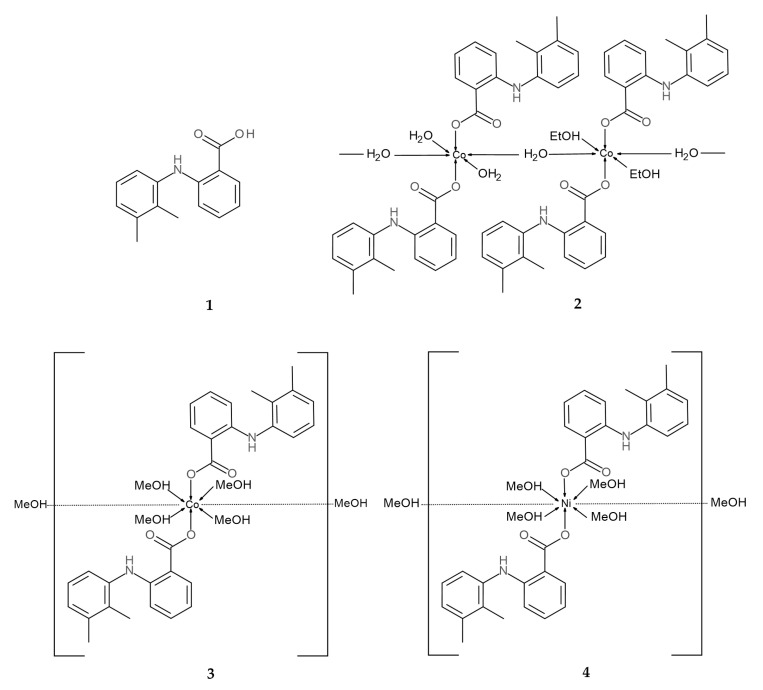
Structural schemes of **1**–**4**.

The synthesis, spectroscopic properties, and thermal stability of six divalent mefenamato complexes Mn(mef)_2_∙3H_2_O **5**, Co(mef)_2_∙2H_2_O **6**, Ni(mef)_2_∙2H_2_O **7**, Cu(mef)_2_∙2H_2_O **8**, Zn(mef)_2_∙2H_2_O **9**, and Cd(mef)_2_∙2H_2_O **10** were described by us previously [40]. In this contribution, we report the successful crystallization and crystal structures of three mefenamato complexes: **2**–**4** (Scheme 1). Furthermore, the high quality and low temperature structure of **3** was also determined and compared to the already published by Dimiza et al. structure of [Co(mef)_2_(MeOH)_4_]∙2MeOH [33]. The latter is characterized by exceptionally high uncertainty ellipsoids of oxygen atoms directly linked to the central cobalt ions. Therefore, we redetermined the structure of **3** through a single crystal X-ray analysis. The high quality data, collected at a temperature T = 100 K, allowed us to define and refine all disordered atoms and provided precise atomic coordinates further used in this work. In addition, the spectral, thermal, antioxidant, and antimicrobial properties of **1**–**4** were determined and compared to the series of **5**–**10**.

## 2. Results and Discussion

Compounds **2**, **3**, and **4** are air stable at room temperature. They are highly soluble in polar organic solvents like DMF, DMSO, methanol, and ethanol. Their properties were thoroughly investigated by the FAAS, EA, FTIR, and TGA methods augmented by antimicrobial and antioxidant studies; see Table 1. The crystal structures of **2**–**4** were determined by the single crystal X-ray diffraction technique; see Table 2. The crystals of **3** and **4** are isomorphic. The analytical data were in good agreement with crystal structures of all complexes.

### 2.1. Description of Crystal Structures

#### 2.1.1. Crystal Structure of **2**

**Figure 1:**
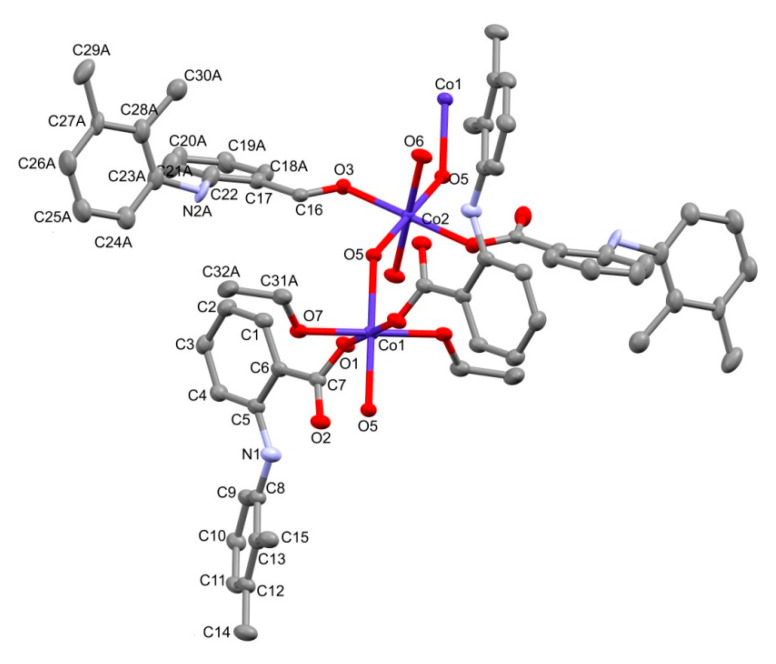
The crystal structure of **2.** Hydrogen atoms are omitted for clarity. Displacement ellipsoids are drawn at the 50% probability level. Only the asymmetric unit is labeled, and the major component of disordered atoms are display for clarity.

The molecular structure of complex **2** is presented in Figure 1. It crystallizes in the triclinic space group P$\bar{1}$ with two symmetrically independent cobalt ions, namely Co1 and Co2, placed in a special positions on inversion centers. Therefore, the single asymmetric unit contains half Co1 and half Co2 ions, two mefenamato moieties, two coordinated waters, and a single coordinated ethanol molecule. Both Co(II) ions are six coordinated, adopt octahedral environments, and share a single apical water O5. This arrangement propagates along the [100] direction, thus leading to a 1-D polymeric structure with a Co1–O5–Co2 repetitive unit (Figure S1). Similar supramolecular motifs were recently identified and published by us [2]. 

Both mefenamato ligands are monodentate with coordinating carboxylate O1 and O3 atoms placed in equatorial positions of Co1 and Co2, respectively. Ethanol O7, as in Co1, and water O6, as in Co2, complete cobalt coordination spheres. The two 2-((2,3-dimethylphenyl)amino)benzoate moieties adopt twisted conformations. The dihedral angles between phenyl rings are 82.65(16) and 38.55(11)° for the species coordinated to Co1 and Co2, respectively. The apical Co1–O5 and Co2–O5 bonds distances are almost identical (2.1518(15) and 2.1493(15) Å, respectively) and are longer than Co–O bonds placed in equatorial positions (2.0454(15)-2.0973(17) Å). 

The presented crystal is stabilized by an extensive network of inter- and intramolecular hydrogen bonds (Table 3). In particular, a strong intramolecular interaction is formed between a sole hydrogen at the coordinating water molecule O6 and the carboxylate O1 of a mefenamato moiety. The hydrogen of the ethanol O7 molecule participates in a hydrogen bond with the carboxylate O3. Moreover, the O5 water molecules are hydrogen donors to bifurcated carboxylate acceptors O2 and O4, and the latter oxygen atoms additionally attract hydrogens of neighboring amine groups N1 and N2A, respectively. The resulting chain motif may be described by the Etter’s symbol $C_{4}^{2}\left(9\right)$ [41,42].

#### 2.1.2. Crystal Structure of **3** and **4**

**Figure 2:**
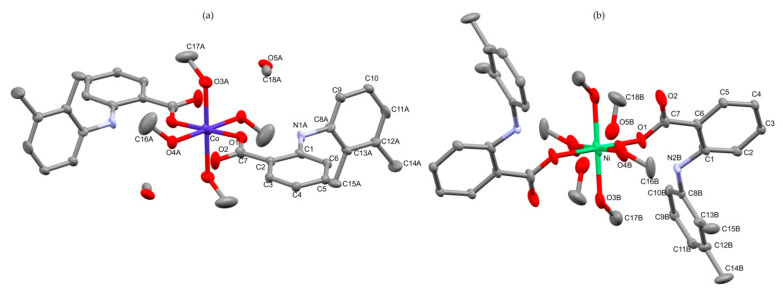
Molecular structures of **3**(a) and **4**(b). Hydrogen atoms are omitted for clarity. Displacement ellipsoids are drawn at the 50% probability level. Only asymmetric units are labeled.

The centrosymmetric, triclinic crystals of **3** and **4** (Figure 2) are quasi-isostructural [43]. Their asymmetric units contain one-half metal ions, a single mefenamato ligand, two coordinated methanol species, and an additional methanol molecule in the outer coordination sphere. The inner coordination spheres of both complexes are quite similar. Octahedral metal ions are placed on inversion centers. They are surrounded by two equatorial mefenamato carboxylate oxygen atoms augmented by two equatorial and two apical methanol molecules. Similarly to **2**, the 2-((2,3-dimethylphenyl)amino)benzoate moieties in **3** and **4** adopt twisted conformations with dihedral angles between phenyl rings equal 74.60(3) and 74.38(18)°, respectively. Apical Co–O3A (2.1220(15) Å) and Ni–O3B (2.113(3) Å) bonds involving methanol species are longer than relevant equatorial bonds (Co–O4A and Ni–O4B; 2.0803(13) and 2.051(2) Å, respectively). The equatorial coordination bonds involving the carboxylate O1 atoms of the mefenamato moieties are slightly shorter (2.0556(8) and 2.0207(8) Å for **3** and **4**, respectively). A structurally similar complex (Mn(mef)_2_(MeOH)_4_) was recently reported by Feng et al. [32]. Following the larger atomic radius of manganese, all bonds within the Mn coordination sphere of this compound are longer than the respective bonds observed in **3** and **4**.

**Figure 3:**
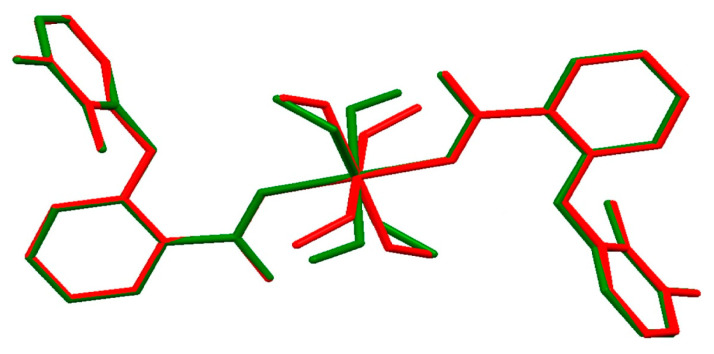
Superposition of structures **3** (red) and **4** (green). Hydrogen atoms and methanol O5 molecules in the outer coordination sphere are omitted for clarity.

The quasi-isostructurality of **3** and **4** is driven by the diverse topologies of the strong hydrogen bonds in both crystals; see Table 4 and Table 5, respectively. The superposition of both structures is presented in Figure 3. In **3**, the carboxylate O2 of the mefanamato moiety is involved in hydrogen bonding with the O4 equatorial methanol molecule. The O5 methanol of the outer coordination sphere is connected to the axial O3. A diverse situation is observed in **4,** where carboxylate O2 interacts with axial O3, while equatorial O4 is involved in H bonding with O5. The latter is a hydrogen donor for contact with carboxylate O2 in either **3** or **4**. The resulting chain may be characterized by motif $C_{3}^{3}\left(7\right)$ (Figure S2). The conformation of mefanamato moieties in all investigated complexes **2**–**4** is stabilized by the intramolecular interactions of the amine nitrogen atoms with carbonyl groups. 

#### 2.1.3. Crystal Packing Analysis of **2–4**

**Figure 4:**
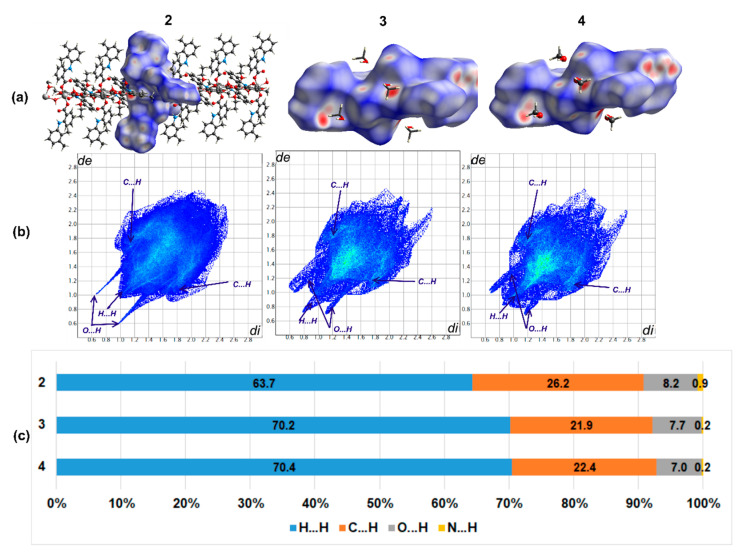
(**a**) Hirshfeld surfaces generated for the mer unit of **2** and the single molecules of **3** and **4**. (**b**) Fingerprint plots generated for respective moieties. Labels indicate regions of specific interatomic interactions. (**c**) The percentage contributions to the Hirshfeld surface areas for intermolecular contacts.

The molecular shapes of structures **2**–**4** were visualized as Hirshfeld surfaces (HSs) (Figure 4). In **2**, the mer unit of infinite polymeric chain was selected for the HS visualization. Relevant fingerprint plots (FPs) derived from the HSs were generated for each structure to characterize the propensity of these species to form particular intermolecular contacts. Crystal packing arrangements in all structures may be defined by three types of contacts, namely H^…^H, C^…^H, and O^…^H. Additionally, the fingerprint analysis revealed the limited presence of N^…^H contacts, as indicated by their contributions below 1%. Due to the polymeric character of **2**, the H^…^H contacts contribution of 63.7% is notably lower than that in **3** and **4**. The latter approach 70.2% and 70.4%, respectively. The FPs of all systems feature symmetrical wings that visualize C^…^H contacts and follow the C–H^…^π and O–H^…^π interactions. The contribution of C^…^H contacts in the crystal packing is visibly higher for polymeric structure **2** (26.2%) than those in **3** and **4** (21.9% and 22.4%, respectively). Similarly, the O^…^H contacts contributions over all structures range from 7.0% to 8.2%. These contacts are depicted as sharp spikes for **2**, while for **3** and **4**, those spikes are slightly bold. The latter presumable follow the methanol molecule disorder in the crystal structures of **3** and **4**. Interestingly, a difference in the intermolecular hydrogen bond topology is also apparent in the O^…^H contact contribution. Structure **3**, where axial O3 is a hydrogen donor for the outer O5 methanol, is characterized by an O^…^H contacts contribution of 7.0%. The equatorial position of the relevant hydrogen bond donor in **4** results in an increase of the O^…^H contacts’ contribution to 7.7%.

The fingerprint analysis showed that supramolecular systems of **2**–**4** are defined by a relatively similar set of contacts. The most distinguished is a polymeric system in **2**. Nonetheless, the quantitative analysis of intermolecular contacts helped to expose the subtle differences in supramolecular assemblies, as observed in the quasi-isostructural systems of **3** and **4**. 

### 2.2. Infrared Spectra of **2–4**

All complexes exhibit sharp bands in the range of 3290–3305 cm^−1^ and weak broad absorption bands in the region of 2900–3200 cm^−1^ associated with the ν(NH) and ν(OH) stretching vibrations, respectively (Figure S3). Additionally, bands at 3610–3630 cm^−1^ for **3** and **4** are assigned to the ν(OH) stretching vibration of methanol molecules. The characteristic bands originated from carboxylate group appear in the range of 1350–1700 cm^−1^. In particular, sharp absorption peaks appearing at 1610.3, 1604.5, and 1604.5 cm^−1^ correspond to ν_as_(COO^–^) and peaks at 1380.8, 1382.8, and 1384.3 cm^−1^ are assigned to ν_s_(COO^–^) stretching vibrations of carboxylate group for **2**, **3**, and **4**, respectively. The parameter ∆ν=ν_as_(COO^–^) – ν_s_(COO^–^) is larger than that for sodium mefenamate [40], and according to Nakamoto [44], the criteria the coordination mode of carboxylate groups may be described as monodentate.

### 2.3. Thermal Analysis of **2–4**

**Figure 5:**
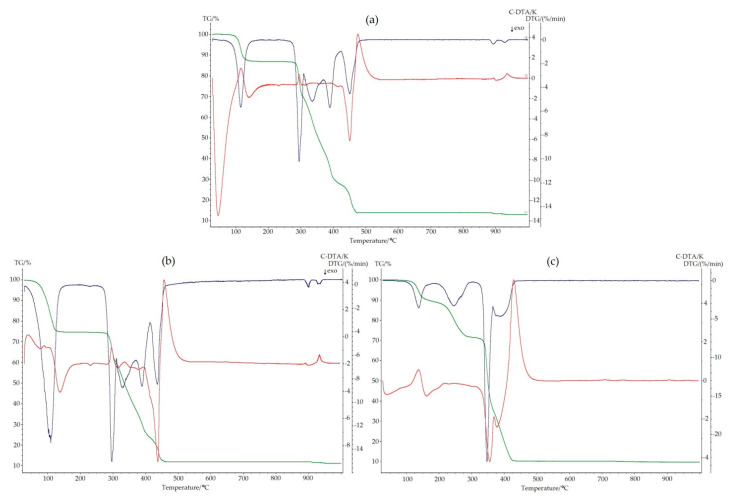
Thermoanalytical curves for **2** (**a**), **3** (**b**), and **4** (**c**). TG is plotted in green, DTG is in blue, while DTA is in red.

The TG/DTG/DTA curves of **2**, **3**, and **4** are summarized in Figure 5. The decomposition pathways are slightly different for each complex. Compound **2** starts to decompose through the concurrent elimination of the ethanol molecule together with two water molecules (mass loss exp. 12.70% and calc. 13.23%) proceeding at the temperature range of 80–250 °C. It is accompanied by endo- and exo-thermic effects on the DTA curve. The next step represents the mass loss (exp. 12.70% and calc. 13.23%) associated with the destruction of two mefenamate ligands at the temperature range of 250–480 °C. The latter leads to the final product Co_3_O_4_ (exp. 12.93% and calc. 12.57%). The corresponding two endo-thermic and one exo-thermic effects were also identified and are shown on the DTA curve. 

The thermal decomposition of **3** and **4** begins with the release of methanol molecules. In **3**, it proceeds in one step, while two distinguished steps were identified for **4**. The weight loss of the former (exp. 26.04% and calc. 26.27%) at 60–260 °C is consistent with the elimination of six methanol molecules altogether. In **4**, two methanol molecules are lost at the temperature range of 100–180 °C (exp. 9.87% and calc. 8.76%), while further four molecules are released at 180–430 °C (exp. 18.13% and calc. 17.52%). The DTA curves exhibit characteristic exo-effects related to those steps. In **4**, both symmetry-related O5 methanols from the outer coordination sphere form weaker hydrogen bonds with the carbonyl O2 than the relevant molecules in **3** (Table 4 and Table 5, respectively). Presumably, the former facilitates a two-step methanol elimination with premature O5 release, as observed in **4**.

Further heating leads to the destruction of both mefenamato moieties in a single step. The experimental weight losses were 62.89% (calc. 62.76%) and 61.77% (calc. 63.51%) for **3** and **4**, respectively. The corresponding exo- and endo-thermal effects are visible on relevant DTA curves. The final products of thermal decompositions are Co_3_O_4_ for **3** and NiO for **4**. Their experimental residual masses (Co_3_O_4_: 11.07%; NiO: 10.28%) were close to calculated values (Co_3_O_4_: 10.97%; NiO: 10.21%).

### 2.4. Antioxidant and Antimicrobial Activities

The antibacterial, antifungal, and antioxidant activities were determined for complexes **2**–**4** and, additionally, **5**–**10** published by us [40]. The above three activities were also analyzed, for the mefenamic acid **1** taken as a reference. 

**Figure 6:**
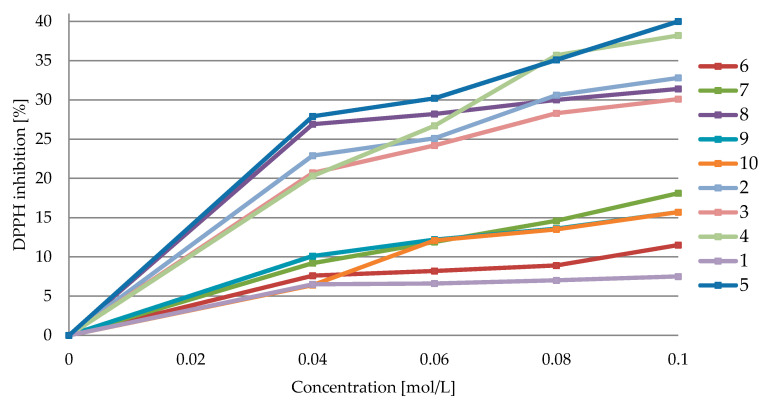
The radical scavenging activities for all complexes. Mefenamic acid (pale purple) is given as a reference.

The investigated complexes exhibited visible RSAs (i.e., higher than that of **1**), with those of **5**, **4**, **2**, and **8** being the highest. The final antioxidant activities are in the order of **5** > **4** > **2** > **8** > **3** > **7** > **9** > **10** > **6** (Figure 6). Similar results were reported by Altun and Suözer [45], who pointed out that the electron withdrawing effect of the metal(II) ion facilitates the release of hydrogen, which further reduces the DPPH radical. 

The results of the antibacterial and antifungal activities are presented in Table 6. Vancomycin (Van) and nystatin (Nys) were used as the standard drugs. Notably, the tested derivatives showed no bioactivity against Gram-negative bacteria (*E. coli* ATCC 25922, *S.* Typhimurium ATCC 14028, *K. pneumoniae* ATCC 13883, *P. aeruginosa* ATCC 9027, and *P. mirabilis* ATCC 12453) with minimal inhibition concentrations (MIC) (MIC 500 – >1000 mg/L). The MIC values for Gram-positive reference bacteria indicated the good (MIC 26–125 mg/L) or moderate (MIC 126–500 mg/L) anti-staphylococcal (*S. aureus* ATCC 25923 and *S. epidermidis* ATCC 12228), anti-micrococcal (*M. luteus* ATCC 10240), and anti-streptococcal (*S. pyogenes* ATCC 19615, *S. pneumonia* ATCC 49619, and *S. mutans* ATCC 25175) activity of the tested compounds [46]. The antibacterial efficiency of the tested derivatives was in the order of **10** > **3** > **2** > **6** > **4** > **9** > **7** > **5** > **8** > **1**. The antifungal bioactivity (*C. albicans* ATCC 2091, *C. glabrata* ATCC 90030, and *C. parapsilosis A*TCC 22019) of the tested compounds was mild or moderate, with that of **10** being the highest. 

## 3. Experimental

### 3.1. Preparation of Complexes and Crystallization

Compounds **2**, **3**, and **4** were synthesized by the dissolution of mefenamic acid (1 mmol) in 50 mL of a freshly precipitated aqueous-ethanol solution (1:1) of NaOH (0.02 mol·L^−1^). Further, sodium mefenamate was heated up to 60 °C and slowly added to an aqueous solution of metal chlorides (0.5 mol in 25 mL). The reaction mixture was kept at 60 °C for 2 hours. After several days, the polycrystalline powders were isolated by filtration, washed with a hot water, and dried in air [40]. 

Single crystals of the **2**, **3**, and **4** complexes suitable for X-ray structure analysis were obtained at room temperature by slow evaporation from the aqueous/ethanol (1:2 *v*/*v*) solution for **2** and from a pure methanol for **3** and **4**. Good quality crystals appeared after two weeks. 

### 3.2. Materials and Methods

Mefenamic acid was purchased from Sigma-Aldrich and used without further purification. Methanol, ethanol, and DPPH (2,2-diphenyl-1-picrylhydrazyl) were purchased from Lab-Scan, and other chemicals were from POCh—Gliwice, Poland. All reagents were chemically pure.

The chemical compositions of **2**–**4** were determined by the elemental analysis (EA) and flame atomic absorption spectrometry (FAAS). Initially, samples (20 mg) were mineralized by the Anton Paar Multiwave 3000 closed microwave system; a mixture of concentrated HNO_3_ and HCl (6:1, *v*/*v*) was applied. Metals concentration were measured by the FAAS with the GBC Scientific Equipment 932 Plus spectrometer. Hydrogen, carbon, and nitrogen contents were measured with the Vario EL III Elemental Analyzer.

The infrared spectra of **2**–**4** were recorded with the Thermo Scientific Nicolet 6700 FTIR spectrometer equipped with liquid nitrogen-cooled MCT (mercury cadmium telluride—HgCdTe) detector. Samples were prepared as KBr pellets and measured over the range of 4000–400 cm^−1^.

The thermal stability and decomposition pathways of **2**–**4** were studied by thermogravimetric techniques. All complexes were measured with a Netzsch TG 209 apparatus. Samples (10 mg) were heated (in ceramic crucibles) up to 1000 °C at a heating rate 10 °C min^−1^ in an air atmosphere. 

The single-crystal X-ray diffraction experiments of **2**–**4** were performed on a Rigaku XtaLAB Synergy Dualflex Pilatus 300K diffractometer. Measurements were conducted using PhotonJet microfocus X-ray source CuK*α* (λ = 1.54184 Å) for **2**, while MoK*α* (λ = 0.71073 Å) radiation was used for **3** and **4**. Crystals were kept at 100.0(1) K during data collection. The data were integrated using the CrysAlisPro software [47]. All structures were solved using the intrinsic phasing method in ShelXT [48] and refined by the full matrix least squares minimization on *F*^2^ with the ShelXL [49] refinement package. The structure of **2** was refined as a two-component twin with a twin scale factor of 0.458(1). The structures of **2**–**4** were notably affected by static disorder, with the mefenamato fragments being disordered over two positions. Relevant occupancy factors were refined to 0.52(3):0.48(3) in **2**, 0.678(3):0.322(3) in **3**, and 0.527(1):0.473(1) in **4**. In **3**, coordinated and non-coordinated methanol molecules were also disordered over two orientations with occupancy factors refined to 0.678(3):0.322(3) and 0.61(2):0.39(2), respectively Similarly, in **4**, methanol molecules were refined over 2 positions, thus resulting in an occupancy factor of 0.591(3):0.409(3). In all structures, the sums of occupancies of relevant sites were set equal to 1 and refined using free variables. The PART instruction was applied to exclude bonding between equivalent disordered atoms. The anisotropic displacement parameters of neighboring disordered atoms were restrained using the SIMU and RIGU procedures in ShelXL. In **2** and **4**, a few disordered atoms were additionally fixed with the EADP instruction. The geometries of disordered fragments were restrained using the DFIX and SADI commands, while O–H and N–H hydrogen atoms were located in difference Fourier maps, C–H hydrogens were generated geometrically using the HFIX command, and a riding model was applied for the refinement. Molecular plots and packing diagrams were drawn using Mercury [50]. Geometry parameters were computed with PLATON [51].

The CIF file for **2**–**4** is available from the Cambridge Crystallographic Data Centre (CCDC) (deposition numbers CCDC: 1989350, 1989353, and 1989354, respectively). Hirshfeld surfaces (HSs) were generated using the CrystalExplorer17 program. Molecular geometries were the same as in crystal structures. For molecular fragments where crystallographic disorder was identified, the major components were only considered. The distances from the HS to the nearest atom interior and exterior to the surface (di and de, respectively) were calculated and plotted as scattergrams [52]. A quantitative decomposition analysis of atom-to-surface contacts was calculated as a percentage of the points in the Hirshfeld surface with di and de for specific atom pairs. 

The radical scavenging activities (RSAs) of **1**–**10** were evaluated by measuring scavenging ability of the DPPH free radical. The solutions were prepared as following: 0.5 mL methanolic solutions of the complexes (0.04, 0.06, 0.08, and 0.1 mol/L) were mixed with the DPPH methanolic solution (60 μM and 0.5 mL) in the dark. The samples were incubated at 25 °C for 30 min in the dark to reach equilibrium before the measurement. The radical scavenging ability of compounds was calculated using the equation: I% = (1 − A_s_/A_0_) × 100, where A_0_ is the absorbance of the sample at 0 min and A_S_ is the absorbance of the sample at 30 min. The RSA experiment was repeated in triplicate. The average values were noted. 

The antibacterial and antifungal activities were screened for **1**–**10** via the micro-dilution broth method according to the European Committee on Antimicrobial Susceptibility Testing (EUCAST) using a Mueller–Hinton broth and a Mueller–Hinton broth with 5% lysed sheep blood for the growth of non-fastidious and fastidious bacteria, respectively, or RPMI with MOPS for the growth of fungi. The minimal inhibitory concentrations (MIC) of the tested derivatives were evaluated for the panel of the reference microorganisms from the American Type Culture Collection (ATCC), including Gram-negative bacteria (*Escherichia coli* ATCC 25922, *Salmonella* Typhimurium ATCC14028, *Klebsiella pneumoniae* ATCC 13883, *Pseudomonas aeruginosa* ATCC 9027, and *Proteus mirabilis* ATCC 12453), Gram-positive bacteria (*Staphylococcus aureus* ATCC 25923, *Staphylococcus epidermidis* ATCC 12228, *Micrococcus luteus* ATCC 10240, *Enterococcus faecalis* ATCC 29212 *Bacillus subtilis* ATCC 6633, *Bacillus cereus* ATCC 10876*, Streptococcus pyogenes* ATCC 19615, *Streptococcus pneumoniae* ATCC 49619, and *Streptococcus mutans* ATCC 25175), and fungi (*Candida albicans* ATCC 10231, *Candida parapsilosis* ATCC 22019, and *Candida glabrata* ATCC 90030). 

The complexes dissolved in DMSO were first diluted to the concentration of 1000 mg/L in an appropriate broth medium recommended for bacteria or yeasts. Then, using the same media, serial two-fold dilutions were made in order to obtain final concentrations of the tested derivatives ranged from 1.95 to 1000 mg/L. The sterile 96-well polystyrene microtitrate plates (Nunc, Denmark) were prepared by dispensing 200 µL of the appropriate dilution of the tested derivatives in broth medium per well. The inocula were prepared with fresh microbial cultures in sterile 0.85% NaCl to match the turbidity of 0.5 McFarland standard, and 2 μL were added to wells to obtain a final density of 1.5 × 10^6^ CFU/mL for bacteria and 5 × 10^4^ CFU/mL for yeasts; CFU—colony forming units. After incubation (bacterial strains—35 °C for 24 h; yeast strains—30 °C for 48 h), the MICs were visually assessed as the lowest concentration of the extracts showing the complete growth inhibition of the reference microbial strains. An appropriate DMSO control (at a final concentration of 10%), a positive control (containing inoculum without the tested derivatives), and a negative control (containing the tested derivatives without inoculum) were included on each microplate. Minimal bactericidal concentration (MBC) or minimal fungicidal concentration (MFC) was determined by subculturing 5 μL of the microbial culture from each well that showed growth inhibition, from the last positive one and from the growth control onto the recommended agar plates. The plates were incubated at 35 °C for 24 h, and the MBC/MFC was defined as the lowest concentration of the extracts without the growth of microorganisms. Each experiment was repeated in triplicate. The highest MIC value was noted [53]. 

## 4. Conclusions

In summary, three metal complexes of cobalt and nickel with mefenamato ligand were characterized by X-ray structure analysis combined with elemental and thermal analyses that were further augmented by spectroscopic and biological activity studies. The structure of **3** and **4** are stabilized by strong intermolecular hydrogen bonds of diverse topologies, thus yielding quasi-isostructural arrangements. Their crystal packing formation is reflected by the Hirshfeld surface analysis of intermolecular contacts. The latter shows subtle differences in the O^…^H contacts contributions, which are closely related to diverse hydrogen bonds topologies and thermal decomposition patterns, as observed in **3** and **4**. In particular, the higher contribution of O^…^H contacts prompts a single step of methanol molecule elimination. On the contrary, a two-stage elimination can be observed in **4**. 

The antibacterial, antifungal, and antioxidant activities were determined for **2**–**4** and additionally for six complexes with mefenamic acid, namely **5**–**10**, the syntheses of which were recently published by us. All investigated complexes showed an antioxidant activity higher than that of mefenamic acid. The most promising antioxidant scavengers were **5** and **4**, with maximum actions of 42.0% and 38.2%, respectively. Additionally, the tested compounds exhibited moderate to strong activity against Gram-positive rods. Compounds **10**, **3**, **2**, and **6** were the most promising antibacterial agents. However, only **10** exhibited a significant antifungal bioactivity.

## Figures and Tables

**Table 1:** Analytical data for **2**–**4**.

No.	Compounds	Empirical Formula	Color	Formula Weight (g/mol)	Elemental Analysis, Found (calc.) (%)			
					C	H	N	M *
**2**	[Co_2_(mef)_4_(EtOH)_2_(H_2_O)_4_]	C_64_H_74_Co_2_N_4_O_14_	pale pink	1241.16	62.21 (61.93)	5.87 (6.01)	4.87 (4.51)	9.96 (9.50)
**3**	[Co(mef)_2_(MeOH)_4_]∙2MeOH	C_36_H_52_CoN_2_O_10_	pink	731.72	59.88 (59.09)	7.02 (7.16)	3.27 (3.83)	8.78 (8.05)
**4**	[Ni(mef)_2_(MeOH)_4_]∙2MeOH	C_36_H_52_NiN_2_O_10_	pale green	731.50	59.58 (59.11)	7.61 (7.17)	3.97 (3.83)	8.48 (8.02)

* M represents Co in **2** and **3** or Ni in **4**.

**Table 2:** Crystal data and structure refinement parameters for **2**–**4**.

	2	3	4
T (K)	100.0	100	100
Crystal system	triclinic	triclinic	triclinic
Space group	P$\bar{1}$	P$\bar{1}$	P$\bar{1}$
a (Å)	7.80610(10)	7.6291(2)	7.6176(2)
b (Å)	13.0847(4)	7.8052(2)	7.8118(4)
c (Å)	15.7219(5)	15.7376(3)	15.7014(3)
α (°)	105.091(3)	89.971(2)	89.966(3)
β (°)	97.760(2)	80.7510(10)	80.276(2)
γ (°)	97.208(3)	87.376(3)	87.349(3)
*V* (Å^3^)	1514.45(7)	923.95(4)	919.91(6)
Z	2	1	1
ρ_calc_ (g/cm^3^)	1.363	1.315	1.320
F(000)	654	389	390
Radiation	CuKα (λ = 1.54184)	MoKα (λ = 0.71073)	MoKα (λ = 0.71073)
2*θ* range (°)	5.914–148.962	5.224–61.012	5.432–63.01
Reflections collected	6987	64201	45789
Independent reflections	6134	5622	6094
Goodness-of-Fit (GOF)	1.050	1.089	1.044
R [I ≥ 2σ (I)]	0.0388	0.0347	0.0368
*w*R_2_ [I ≥ 2σ (I)]	0.1027	0.0941	0.0953

**Table 3:** Hydrogen bond geometry in **2** (Å,°).

D-H^…^A	D-H	H^…^A	D^…^A	D-H^…^A
O6-H6A^…^O1 ^a^	0.83(2)	1.85(2)	2.672(2)	170(3)
O7-H6^…^O3	0.83(3)	1.84(3)	2.670(2)	174(3)
O5-H5B^…^O2	0.85(2)	1.74(2)	2.560(2)	161(2)
O5-H5A^…^O4 ^b^	0.835(18)	1.723(19)	2.541(2)	166(2)
N1-H1^…^O2	0.90(3)	1.87(3)	2.661(3)	145(3)
N2A-H2A^…^O4	0.84(6)	1.94(5)	2.58(2)	132(6)

Symmetry codes: (^a^) −1+x, y, z; (^b^) 1−x, 1−y, −z.

**Table 4:** Hydrogen bond geometry in **3** (Å,°).

D-H^…^A	D-H	H^…^A	D^…^A	D-H^…^A
N1A-H1A^…^O1	0.813(18)	1.95(2)	2.552(5)	130(2)
O3A-H3A^…^O5A	0.72(3)	2.13(3)	2.835(5)	168(3)
O4A-H4A^…^O2	0.92(3)	1.72(3)	2.6045(16)	162(3)
O5B-H5B^…^O2 ^a^	0.82(6)	2.04(6)	2.676(11)	134(5)

Symmetry codes: (^a^) −1+x, y, z.

**Table 5:** Hydrogen bond geometry in **4** (Å,°).

D-H^…^A	D-H	H^…^A	D^…^A	D-H^…^A
N2B-H2B^…^O1	0.83(2)	1.83(3)	2.521(6)	133(4)
O3A-H3A^…^O2 ^a^	0.84(2)	1.772(19)	2.5989(17)	166(3)
O4A-H4A^…^O5A	0.84(3)	1.86(3)	2.663(5)	161(4)
O5A-H5A^…^O2 ^b^	0.83(7)	2.12(7)	2.732(6)	131(8)

Symmetry codes: (^a^) 1-x, 1-y, 1-z; (^b^) -1+x, y, z.

**Table 6:** The antibacterial and antifungal activities.

**Bacteria**	**MIC (mg/L)**										
	**1**	**2**	**3**	**4**	**5**	**6**	**7**	**8**	**9**	**10**	**Van**
*S. aureus* ATCC 25923	1000	125	125	125	250	125	125	250	125	62.5	0.98
*S. epidermidis* ATCC 12228	>1000	250	250	250	500	250	250	250	125	62.5	0.98
*M. luteus* ATCC 10240	62.5	125	62.5	125	250	62.5	125	62.5	125	3.9	0.12
*E. faecalis* ATCC 29212	>1000	1000	>1000	500	1000	500	500	1000	500	15.6	1.95
*B. subtilis* ATCC 6633	62.5	125	62.5	125	250	125	125	125	125	7.8	0.24
*B. cereus* ATCC 10876	62.5	62.5	62.5	125	125	62.5	125	62.5	62.5	62.5	0.98
*S, pyogenes* ATCC 19615	125	125	62.5	62.5	250	31.3	125	125	250	31.3	0.24
*S. pneumoniae* ATCC 49619	125	62.5	62.5	62.5	250	31.3	125	125	125	7.8	0.24
*S. mutans* ATCC 25175	500	125	125	250	>1000	125	50	250	250	15.6	0.98
**Yeasts**	**MIC (mg/L)**										
	**1**	**2**	**3**	**4**	**5**	**6**	**7**	**8**	**9**	**10**	**Nys**
*C. albicans* ATCC 102231	1000	1000	1000	1000	500	1000	250	1000	500	250	0.48
*C. parapsilosis* ATCC 22019	1000	1000	1000	1000	500	500	250	500	500	15.6	0.24
*C. glabrata* ATCC 90030	1000	500	500	1000	250	500	500	1000	1000	125	0.24

## Supplementary Materials

The supplementary material includes: Polymeric chain running along the [100] axis in **2** (Figure S1); Fragment of the intermolecular chain along [100] stabilized by hydrogen bonds, as in **3**(a) and **4**(b) (Figure S2); FTIR spectra of **2**–**4** (Figure S3). Bond distances for **2**, **3**, and **4** (Tables S1, S4, and S7, respectively); Bond angles for **2**, **3**, and **4** (Tables S2, S5 and S8, respectively); Torsion angles for **2**, **3** and **4** (Tables S3, S6 and S9, respectively).

## References

[1] Krawczyk M.S., Majerz I. (2019). The Na-O Bond In sodium fenamate. Acta Cryst..

[2] Gacki M., Kafarska K., Wolf W.M. (2019). A supramolecular polymeric chain in the cobalt(II) complex with diclofenac: Synthesis, crystal structure, spectroscopic, thermal and antioxidant activity. J. Coord. Chem..

[3] Tarushi A., Geromuchalos G.D., Lafazanis K., Raptopoulou C.P., Psycharis V., Lalioti L., Pantazaki A.A., Kessissoglou D.P., Tangoulis V., Psomas G. (2018). A step-ladder manganese(III) metallacrown hosting mefenamic acid and a manganese(II)–mefanamato complex: Synthesis, characterization and cytotoxicactivity. N. J. Chem..

[4] De Groot D.J.A., de Vries E.G.E., Groen H.J.M., de Jong S. (2007). Non-steroidal anti-inflammatory drugs to potentiate chemotherapy effects: From lab to clinic. Crit. Rev. Oncol. Hematol..

[5] Orido T., Fujino H., Hasegawa Y., Toyomura K., Kawashima T., Murayama T. (2008). Indomethacin Decreases Arachidonic Acid Uptake in HCA-7 Human Colon Cancer Cells. J. Pharmacol. Sci..

[6] Altay A., Caglar S., Ceglar B., Sahin O. (2018). Synthesis, structural, thermal elucidation and in vitro anticancer activity of novel silver(I) complex with non-steroidal anti-inflammatory drugs diclofenac and mefenamic acid including picoline derivatives. Polyhedron.

[7] Herendeen J.M., Lindley C. (2003). Use of NSAIDs for the chemoprevention of colorectal cancer. Ann. Pharmacother..

[8] Rao C.V., Reddy B.S. (2004). NSAIDs and chemoprevention. Curr. Cancer Drug Targets.

[9] Bernstein J. (2002). Polymorphism in Molecular Crystals.

[10] Salbego P.R.S., Bender C.R., Orlando T., Moraes G.A., Copetti J.P.P., Weimer G.H., Bonacorso H.G., Zanatta N., Hoerner M., Martins M.A.P. (2019). Supramolecular similarity in polymorphs: Use of similarity indices (I^x^). ACS Omega.

[11] Resnati G., Boldyreva E., Bombicz P., Kawano M. (2015). Supramolecular interactions in the solid state. IUCrJ.

[12] Ranjan S., Devarapalli R., Kundu S., Saha S., Deolka S., Vangala V.R., Reddy C.M. (2020). Isomorphism: ‘molecular similarity of crystal structure similarity’ in multicompponent forms of analgesic drugs tolfenamic and mefenamic acid. IUCrJ.

[13] Bombicz P. (2017). The way from isostructurality to polymorphism. Where are the borders? The role of supramolecular interactions and crystal symmetries. Crystallogr. Rev..

[14] Scherrer R.A., Arbor A. (1960). Anthranilic Acid Derivatives. U.S. Patent.

[15] Whitehouse M.W. (2005). Drugs to treat inflammation: A historical introduction. Curr. Med. Chem..

[16] Smolková R., Smolko L., Zeleňák V., Kuchárc J., Gyepes R., Talian I., Sabo J., Biščáková Z., Rabajdová M. (2019). Impact of the central atom on human genomic DNA and human serum albumin binding properties in analogous Zn(II) and Cd(II) complexes with mefenamic acid. J. Mol. Struct..

[17] Adam A., Schrimpl L., Schmidt P.C. (2000). Some physicochemical properties of mefenamic acid. Drug Dev. Ind. Pharm..

[18] Batool S.S., Gilani S.R., Zainab S.S., Tahir M.N., Harrison W.T.A., Syed Q., Mazhar S. (2019). Synthesis and structural characterization of a monomeric mixed ligand copper(II) complex involving N, N,N’,N’-tetramethylethylenediamine and mefenamate. J. Struct. Chem..

[19] Joo Y., Kim H.-S., Woo R.-S., Hyoung Park H., Shin K.-Y., Lee J.-P., Chang K.-A., Kim S., Suh Y.-H. (2006). Mefenamic acid shows neuroprotective effects and improves cognitive impairment in in vitro and in vivo alzheimer’s disease models. Mol. Pharmacol..

[20] SeethaLekshmi S., Guru Row T.N. (2012). Conformational polymorphism in a non-steroidal anti-inflammatory drug, mefenamic acid. Cryst. Growth Des..

[21] Abbas N., Oswald I.D.H., Pulham C.R. (2017). Accessong mefenamic acid form II through high-pressure recrystallisation. Pharmaceutics.

[22] Aguiar A.J., Zelmer J.E. (1969). Dissolution behavior of polymorphs of chloramphenicol palmitate and mefenamic acid. J. Pharm. Sci..

[23] Romero S., Escalera B., Bustamante P. (1999). Solubility behavior of polymorphs I and II of mefenamic acid in solvent mixtures. Int. J. Pharm..

[24] McConnell J., Company F.Z. (1976). N-(2,3-xylyl) anthranilic acid, C_15_H_15_NO_2_. Mefenamic acid. Cryst. Struct. Commun..

[25] Lee E.H., Byrn S.R., Carvajal M.T. (2006). Additive-induced metastable single crystal of mefenamic acid. Pharm. Res..

[26] Amidon G.L., Lennernas H., Shah V.P., Crison J.R.A. (1995). A theoretical basis for a bio-pharmaceutical drug classification: The correlation of in vitro drug product dissolution and in vivo bioavailability. Pharm. Res..

[27] Reiss A., Cioatera N., Chifiriuc M.C., Munteanu G., Ganescu A., Dabuleanu I., Avram G., Spinu I.C., Rotaru P.J. (2018). New biologically active mixed-ligand Co(II) and Ni(II) complexes of enrofloxacin. Therm. Anal. Calorim..

[28] Mewis R., Archibald S.J. (2010). Biomedical applications of macrocyclic ligand complexes. Coord. Chem. Rev..

[29] Badea M., Pătraşcu F., Cerc Korošec R., Bukovec P., Raita M., Chifiriuc M.C., Măruţescu L., Bleotu C., Velescu B., Marinescu D. (2014). Thermal, spectral, magnetic and biologic characterization of new Ni(II), Cu(II) and Zn(II) complexes with a hexaazamacrocyclic ligand bearing ketopyridine moieties. J. Therm. Anal. Calorim..

[30] Kovala-Demertzi D., Staninska M., Garcia-Santos I., Castineiras A., Demertzis M.A. (2011). Synthesis, crystal structures and spectroscopy of meclofenamic acid and its metal complexes with manganese(II), copper(II), zinc(II) and cadmium(II). Antiproliferative and superoxide dismutase activity. J. Inorg. Biochem..

[31] Kovala-Demertzi D., Dokorou V., Primikiri A., Vargas R., Silvestru C., Russo U., Demertzisa M.A. (2009). Organotin meclofenamic complexes: Synthesis, crystal structures and antiproliferative activity of the first complexes of meclofenamic acid—Novel anti-tuberculosis agents. J. Inorg. Biochem..

[32] Feng J., Du X., Liu H., Sui X., Zhang C., Tang Y., Zhang J. (2014). Manganese-mefenamic acid complexes exhibit high lipoxygenase inhibitory activity. Dalton Trans..

[33] Dimiza F., Papadopoulos A.N., Tangoulis V., Psycharis V., Raptopoulou C.P., Kessissoglou D.P., Psomas G. (2010). Biological evaluation of non-steroidal anti-inflammatory drugs-cobalt(ii) complexes. Dalton Trans..

[34] Tottaa X., Papadopoulou A.A., Hatzidimitriou A.G., Papadopoulos A., Psomas G. (2015). Synthesis, structure and biological activity of nickel(II) complexes with mefenamato and nitrogen-donor ligands. J. Inorg. Biochem..

[35] Facchin G., Torre M.H., Kremer E., Piro O.E., Baran E.J. (1998). Crystal structure and spectroscopic behaviour of a binuclear copper(II) complex of mefenamic acid and dimethylsulfoxide. Z. Nat..

[36] Sharma R.P., Kumar S., Venugopalan P., Ferretti V., Tarushi A., Psomas G., Witwicki M. (2016). New copper(ii) complexes of the anti-inflammatory drug mefenamic acid: A concerted study including synthesis, physicochemical characterization and their biological evaluation. RSC Adv..

[37] Dimiza F., Fountoulaki S., Papadopoulos A.N., Kontogiorgis C.A., Tangoulis V., Raptopoulou C.P., Psycharis V., Terzis A., Kessissoglou A.P., Psomas G. (2011). Non-steroidal antiinflammatory drug–copper(ii) complexes: Structure and biological perspectives. Dalton Trans..

[38] Tarushi A., Karaflou Z., Kljun J., Turel I., Psomas G., Papadopoulos A.N., Kessissoglou D.P. (2013). Antioxidant capacity and DNA-interaction studies of zinc complexes with a non-steroidal anti-inflammatory drug, mefenamic acid. J. Inorg. Biochem..

[39] Dokorou V., Ciunik Z., Russo U., Kovala-Demertzi D. (2001). Synthesis, crystal structures and spectroscopic studies of diorganotin derivatives with mefenamic acid. Crystal and molecular structures of 1,2:3,4-di-μ_2_-2-[(2,3-dimethylphenyl)amino]-benzoato-*O*,*O*-1,3-bis-2-[(-[(2,3-dimethylphenyl)amino]benzoato-*O*-1,2,4:2,3,4-di-μ_3_-oxo-tetrakis[di-methyltin(IV)] and 1,2:3,4-di-μ_2_-2-[(-[(-[(2,3-dimethylphenyl)amino]-benzoato-*O*,*O*-1,3-bis-2-[(-[(-[(2,3-dimethylphenyl)amino]benzoato-*O*-1,2,4:2,3,4-di-μ_3_-oxo-tetrakis[di-*n*-butyltin(IV)]. J. Organomet. Chem..

[40] Kafarska K., Gacki M., Wolf W.M. (2017). Synthesis, spectroscopic, and thermal investigations of metal complexes with mefenamic acid. J. Chem..

[41] Bernstein J., Davis R., Shimoni L., Chang N.-L. (1995). Patterns in hydrogen bonding: Functionality and graph set analysis in crystals. Angew. Chem. Int. Ed..

[42] Etter M.C. (1990). Encoding and decoding hydrogen-bond patterns of organic compounds. Acc. Chem. Res..

[43] Dey D., Thomas S.P., Spackman M.A., Chopra D. (2016). ‘Quasi-isostructural polymorphism’ in molecular crystals: Inputs from interaction hierarchy and energy frameworks. Chem. Commun..

[44] Nakamoto K. (2009). Infrared and Raman Spectra of Inorganic and Coordination Compounds.

[45] Altun O., Suözer M. (2017). Synthesis, spectral analysis, stability contants, antioxidant and biological activities of Co(II), Ni(II) and Cu(II) mixed ligand complexes of nicotinamide, theophylline and thiocyanate. J. Mol. Struct..

[46] O’Donnell F., Smyth T.J.P., Ramachandran V.N., Smyth W.F. (2009). A study of the antimicrobial activity of selected synthetic and naturally occurring quinolines. Int. J. Antimicrob. Agents.

[47] (2017). CrysAlis Pro.

[48] Sheldrick G.M. (2015). SHELXT-Integrated space-group and crystal-structure determination. Acta Crystallogr. Sect. C Struct. Chem..

[49] HüBschle C.B., Sheldrick G.M., Dittrich B. (2011). ShelXle: A Qt graphical user interface for SHELXL. J. Appl. Cryst..

[50] Macrae C.F., Bruno I.J., Chisholm J.A., Edgington P.R., Mccabe P., Pidcock E., Rodriguez-Monge L., Taylor R., Van De Streek J., Wood P.A. (2008). Mercury: Visualization and analysis of crystal structures. J. Appl. Cryst..

[51] Spek A.L. (2009). Structure validation in chemical crystallography. Acta Crystallogr. Sect. D Biol. Crystallogr..

[52] McKinnon J.J., Jayatilaka D., Spackman M.A. (2007). Towards quantitative analysis of intermolecular interactions with Hirshfeld surfaces. Chem. Commun..

[53] Żesławska E., Korona-Głowniak I., Szczesio M., Olczak A., Żylewska A., Tejchman W., Malm A. (2017). Structural analysis and antimicrobial activity of 2[1H]-pyrimidinethione/selenone derivatives. J. Mol. Struct..

