# Supplementary material for: Quasi-Isostructural Co(II) and Ni(II) Complexes with Mefenamato Ligand: Synthesis, Characterization, and Biological Activity
Source: Molecules. 2020 Jul 7;25(13):3099. doi: 10.3390/molecules25133099 (PMC7412345; doi:10.3390/molecules25133099)

checkCIF/PLATON report

Structure factors have been supplied for datablock(s) Co_mef_th_21_05_twin1_hklf4

THIS REPORT IS FOR GUIDANCE ONLY. IF USED AS PART OF A REVIEW PROCEDURE FOR PUBLICATION, IT SHOULD NOT REPLACE THE EXPERTISE OF AN EXPERIENCED CRYSTALLOGRAPHIC REFEREE.

No syntax errors found. CIF dictionary Interpreting this report

Datablock: Co_mef_th_21_05_twin1_hklf4

```
Bond precision:  C-C = 0.0031 A          Wavelength=1.54184

Cell:          a=7.8061(1)        b=13.0847(4)     c=15.7219(5)
               alpha=105.091(3)   beta=97.760(2)   gamma=97.208(3)
Temperature:   100 K

                Calculated                Reported
Volume          1514.46(8)                1514.45(7)
Space group     P -1                      P -1
Hall group      -P 1                      -P 1
Moiety formula  C32 H38 Co N2 O7          C32 H38 Co N2 O7
Sum formula     C32 H38 Co N2 O7          C32 H38 Co N2 O7
Mr              621.57                    621.57
Dx,g cm-3       1.363                     1.363
Z               2                         2
Mu (mm-1)       4.857                     4.857
F000            654.0                     654.0
F000’           651.19
h,k,lmax        9,16,19                   9,16,19
Nref            6184                      6987
Tmin,Tmax       0.191,0.352               0.477,1.000
Tmin’           0.089

Correction method= # Reported T Limits: Tmin=0.477 Tmax=1.000
AbsCorr = MULTI-SCAN

Data completeness= 1.130          Theta(max)= 74.481

R(reflections)= 0.0388( 6134)     wR2(reflections)= 0.1065( 6987)

S = 1.050                  Npar= 513
```

The following ALERTS were generated. Each ALERT has the format
test-name_ALERT_alert-type_alert-level.
Click on the hyperlinks for more details of the test.

Alert level B

```
PLAT420_ALERT_2_B D-H Without Acceptor       O6       --H6B      .      Please Check
```

Alert level C

```
PLAT911_ALERT_3_C Missing FCF Refl Between Thmin & STh/L=    0.600           2 Report
```

Alert level G

```
PLAT002_ALERT_2_G Number of Distance or Angle Restraints on AtSite         13 Note
PLAT003_ALERT_2_G Number of Uiso or Uij Restrained non-H Atoms ...         33 Report
PLAT004_ALERT_5_G Polymeric Structure Found with Maximum Dimension          1 Info
PLAT171_ALERT_4_G The CIF-Embedded .res File Contains EADP Records          1 Report
PLAT172_ALERT_4_G The CIF-Embedded .res File Contains DFIX Records          4 Report
PLAT176_ALERT_4_G The CIF-Embedded .res File Contains SADI Records          1 Report
PLAT178_ALERT_4_G The CIF-Embedded .res File Contains SIMU Records          4 Report
PLAT187_ALERT_4_G The CIF-Embedded .res File Contains RIGU Records          4 Report
PLAT301_ALERT_3_G Main Residue  Disorder ..............(Resd  1  )        35% Note
PLAT380_ALERT_4_G Incorrectly? Oriented X(sp2)-Methyl Moiety .....        C14 Check
PLAT414_ALERT_2_G Short Intra D-H..H-X       H6       ..H31C            2.11 Ang.
                                                       x,y,z  =      1_555 Check
PLAT794_ALERT_5_G Tentative Bond Valency for Co2       (II)      .       2.02 Info
PLAT811_ALERT_5_G No ADDSYM Analysis: Too Many Excluded Atoms ....          ! Info
PLAT860_ALERT_3_G Number of Least-Squares Restraints .............        442 Note
PLAT870_ALERT_4_G ALERTS Related to Twinning Effects Suppressed ..          ! Info
PLAT912_ALERT_4_G Missing # of FCF Reflections Above STh/L=  0.600         87 Note
```

---

0 **ALERT level A** = Most likely a serious problem - resolve or explain
1 **ALERT level B** = A potentially serious problem, consider carefully
1 **ALERT level C** = Check. Ensure it is not caused by an omission or oversight
16 **ALERT level G** = General information/check it is not something unexpected

0 ALERT type 1 CIF construction/syntax error, inconsistent or missing data
4 ALERT type 2 Indicator that the structure model may be wrong or deficient
3 ALERT type 3 Indicator that the structure quality may be low
8 ALERT type 4 Improvement, methodology, query or suggestion
3 ALERT type 5 Informative message, check

It is advisable to attempt to resolve as many as possible of the alerts in all categories. Often the minor alerts point to easily fixed oversights, errors and omissions in your CIF or refinement strategy, so attention to these fine details can be worthwhile. In order to resolve some of the more serious problems it may be necessary to carry out additional measurements or structure refinements. However, the purpose of your study may justify the reported deviations and the more serious of these should normally be commented upon in the discussion or experimental section of a paper or in the "special_details" fields of the CIF. checkCIF was carefully designed to identify outliers and unusual parameters, but every test has its limitations and alerts that are not important in a particular case may appear. Conversely, the absence of alerts does not guarantee there are no aspects of the results needing attention. It is up to the individual to critically assess their own results and, if necessary, seek expert advice.

Publication of your CIF in IUCr journals

A basic structural check has been run on your CIF. These basic checks will be run on all CIFs submitted for publication in IUCr journals (*Acta Crystallographica*, *Journal of Applied Crystallography*, *Journal of Synchrotron Radiation*); however, if you intend to submit to *Acta Crystallographica Section C* or *E* or *IUCrData*, you should make sure that full publication checks are run on the final version of your CIF prior to submission.

Publication of your CIF in other journals

Please refer to the *Notes for Authors* of the relevant journal for any special instructions relating to CIF submission.

PLATON version of 22/12/2019; check.def file version of 13/12/2019

Datablock Co_mef_th_21_05_twin1_hklf4 - ellipsoid plot

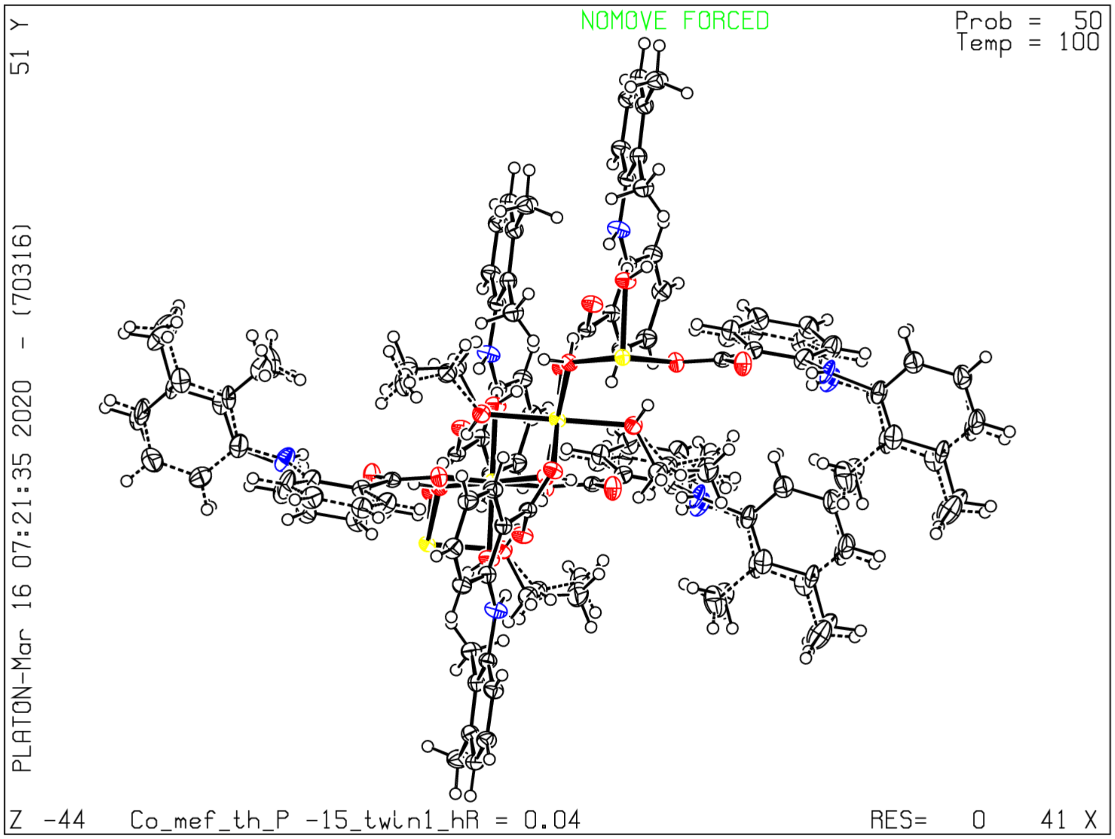

Supplement: Supplementary file 1 [file molecules-25-03099-s001.zip › Structure_2_checkcif.pdf]
