# Supplementary material for: Quasi-Isostructural Co(II) and Ni(II) Complexes with Mefenamato Ligand: Synthesis, Characterization, and Biological Activity
Source: Molecules. 2020 Jul 7;25(13):3099. doi: 10.3390/molecules25133099 (PMC7412345; doi:10.3390/molecules25133099)

checkCIF/PLATON report

Structure factors have been supplied for datablock(s) co_hmef_new

THIS REPORT IS FOR GUIDANCE ONLY. IF USED AS PART OF A REVIEW PROCEDURE FOR PUBLICATION, IT SHOULD NOT REPLACE THE EXPERTISE OF AN EXPERIENCED CRYSTALLOGRAPHIC REFEREE.

No syntax errors found. CIF dictionary Interpreting this report

Datablock: co_hmef_new

Bond precision: C-C = 0.0014 A Wavelength=0.71073

Cell:	a=7.6291(2)	b=7.8052(2)	c=15.7376(3)
	alpha=89.971(2)	beta=80.751(1)	gamma=87.376(3)
Temperature:	100 K		

	Calculated	Reported
Volume	923.95(4)	923.95(4)
Space group	P -1	P -1
Hall group	-P 1	-P 1
Moiety formula	C34 H44 Co N2 O8, 2(C H4 O)	C34 H44 Co N2 O8, 2(C H4 O)
Sum formula	C36 H52 Co N2 O10	C36 H52 Co N2 O10
Mr	731.73	731.72
Dx,g cm-3	1.315	1.315
Z	1	1
Mu (mm-1)	0.522	0.522
F000	389.0	389.0
F000’	389.53	
h,k,lmax	10,11,22	10,11,22
Nref	5624	5622
Tmin,Tmax	0.800,0.876	0.471,1.000
Tmin’	0.770	

Correction method= # Reported T Limits: Tmin=0.471 Tmax=1.000
AbsCorr = GAUSSIAN

Data completeness= 1.000 Theta(max)= 30.506

R(reflections)= 0.0347(5145) wR2(reflections)= 0.0958(5622)

S = 1.089 Npar= 381

The following ALERTS were generated. Each ALERT has the format
test-name_ALERT_alert-type_alert-level.
Click on the hyperlinks for more details of the test.

Alert level C

```
PLAT220_ALERT_2_C NonSolvent Resd 1  C   Ueq(max) / Ueq(min) Range        4.9 Ratio
PLAT222_ALERT_3_C NonSolvent Resd 1  H   Uiso(max)/Uiso(min) Range        6.2 Ratio
PLAT911_ALERT_3_C Missing FCF Refl Between Thmin & STh/L=    0.600          2 Report
```

Alert level G

```
PLAT002_ALERT_2_G Number of Distance or Angle Restraints on AtSite         24 Note
PLAT003_ALERT_2_G Number of Uiso or Uij Restrained non-H Atoms ...         10 Report
PLAT007_ALERT_5_G Number of Unrefined Donor-H Atoms .............           1 Report
PLAT164_ALERT_4_G Nr. of Refined C-H H-Atoms in Heavy-Atom Struct.          2 Note
PLAT172_ALERT_4_G The CIF-Embedded .res File Contains DFIX Records          7 Report
PLAT176_ALERT_4_G The CIF-Embedded .res File Contains SADI Records          2 Report
PLAT177_ALERT_4_G The CIF-Embedded .res File Contains DELU Records          2 Report
PLAT178_ALERT_4_G The CIF-Embedded .res File Contains SIMU Records          2 Report
PLAT187_ALERT_4_G The CIF-Embedded .res File Contains RIGU Records          2 Report
PLAT230_ALERT_2_G Hirshfeld Test Diff for    C10      --C11A     .        6.0 s.u.
PLAT230_ALERT_2_G Hirshfeld Test Diff for    C10      --C11B     .        7.5 s.u.
PLAT232_ALERT_2_G Hirshfeld Test Diff (M-X)  Co       --O4A      .        5.0 s.u.
PLAT301_ALERT_3_G Main Residue  Disorder ..............(Resd  1  )        49% Note
PLAT302_ALERT_4_G Anion/Solvent/Minor-Residue Disorder (Resd  2  )       100% Note
PLAT302_ALERT_4_G Anion/Solvent/Minor-Residue Disorder (Resd  3  )       100% Note
PLAT304_ALERT_4_G Non-Integer Number of Atoms in ..... (Resd  2  )       3.66 Check
PLAT304_ALERT_4_G Non-Integer Number of Atoms in ..... (Resd  3  )       2.34 Check
PLAT380_ALERT_4_G Incorrectly? Oriented X(sp2)-Methyl Moiety .....       C14A Check
PLAT811_ALERT_5_G No ADDSYM Analysis: Too Many Excluded Atoms ....          ! Info
PLAT860_ALERT_3_G Number of Least-Squares Restraints ............         178 Note
PLAT910_ALERT_3_G Missing # of FCF Reflection(s) Below Theta(Min).          1 Note
PLAT933_ALERT_2_G Number of OMIT Records in Embedded .res File ...          1 Note
PLAT978_ALERT_2_G Number C-C Bonds with Positive Residual Density.          9 Info
```

```
 0 ALERT level A = Most likely a serious problem - resolve or explain
 0 ALERT level B = A potentially serious problem, consider carefully
 3 ALERT level C = Check. Ensure it is not caused by an omission or oversight
23 ALERT level G = General information/check it is not something unexpected

 0 ALERT type 1 CIF construction/syntax error, inconsistent or missing data
 8 ALERT type 2 Indicator that the structure model may be wrong or deficient
 5 ALERT type 3 Indicator that the structure quality may be low
11 ALERT type 4 Improvement, methodology, query or suggestion
 2 ALERT type 5 Informative message, check
```

It is advisable to attempt to resolve as many as possible of the alerts in all categories. Often the minor alerts point to easily fixed oversights, errors and omissions in your CIF or refinement strategy, so attention to these fine details can be worthwhile. In order to resolve some of the more serious problems it may be necessary to carry out additional measurements or structure refinements. However, the purpose of your study may justify the reported deviations and the more serious of these should normally be commented upon in the discussion or experimental section of a paper or in the "special_details" fields of the CIF. checkCIF was carefully designed to identify outliers and unusual parameters, but every test has its limitations and alerts that are not important in a particular case may appear. Conversely, the absence of alerts does not guarantee there are no aspects of the results needing attention. It is up to the individual to critically assess their own results and, if necessary, seek expert advice.

Publication of your CIF in IUCr journals

A basic structural check has been run on your CIF. These basic checks will be run on all CIFs submitted for publication in IUCr journals (*Acta Crystallographica*, *Journal of Applied Crystallography*, *Journal of Synchrotron Radiation*); however, if you intend to submit to *Acta Crystallographica Section C* or *E* or *IUCrData*, you should make sure that full publication checks are run on the final version of your CIF prior to submission.

Publication of your CIF in other journals

Please refer to the *Notes for Authors* of the relevant journal for any special instructions relating to CIF submission.

PLATON version of 22/12/2019; check.def file version of 13/12/2019

Datablock co_hmef_new - ellipsoid plot

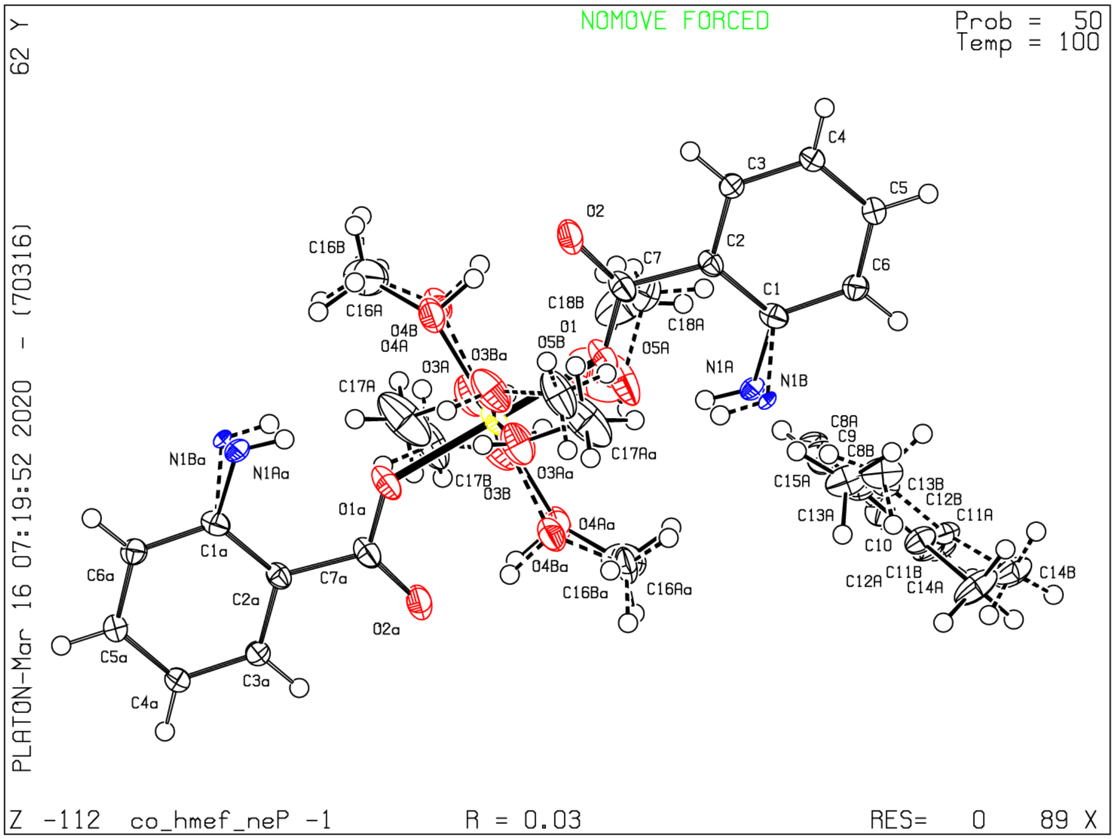

Supplement: Supplementary file 1 [file molecules-25-03099-s001.zip › Structure_3_checkcif.pdf]
