# Supplementary material for: Quasi-Isostructural Co(II) and Ni(II) Complexes with Mefenamato Ligand: Synthesis, Characterization, and Biological Activity
Source: Molecules. 2020 Jul 7;25(13):3099. doi: 10.3390/molecules25133099 (PMC7412345; doi:10.3390/molecules25133099)

checkCIF/PLATON report

Structure factors have been supplied for datablock(s) Ni_mef_1

THIS REPORT IS FOR GUIDANCE ONLY. IF USED AS PART OF A REVIEW PROCEDURE FOR PUBLICATION, IT SHOULD NOT REPLACE THE EXPERTISE OF AN EXPERIENCED CRYSTALLOGRAPHIC REFEREE.

No syntax errors found. CIF dictionary Interpreting this report

Datablock: Ni_mef_1

Bond precision: C-C = 0.0014 A Wavelength=0.71073

Cell:	a=7.6176(2)	b=7.8118(4)	c=15.7014(3)
	alpha=89.966(3)	beta=80.276(2)	gamma=87.349(3)
Temperature:	100 K		

	Calculated	Reported
Volume	919.91(6)	919.91(6)
Space group	P -1	P -1
Hall group	-P 1	-P 1
Moiety formula	C34 H44 N2 Ni O8, 2(C H4 O)	C34 H44 N2 Ni O8, 2(C H4 O)
Sum formula	C36 H52 N2 Ni O10	C36 H52 N2 Ni O10
Mr	731.49	731.50
Dx,g cm-3	1.320	1.320
Z	1	1
Mu (mm-1)	0.585	0.585
F000	390.0	390.0
F000’	390.52	
h,k,lmax	11,11,23	11,11,23
Nref	6141	6094
Tmin,Tmax	0.746,0.878	0.855,1.000
Tmin’	0.730	

Correction method= # Reported T Limits: Tmin=0.855 Tmax=1.000
AbsCorr = MULTI-SCAN

Data completeness= 0.992 Theta(max)= 31.505

R(reflections)= 0.0368(5925) wR2(reflections)= 0.0958(6094)

S = 1.044 Npar= 352

The following ALERTS were generated. Each ALERT has the format
test-name_ALERT_alert-type_alert-level.
Click on the hyperlinks for more details of the test.

Alert level C

```
PLAT220_ALERT_2_C NonSolvent Resd 1  C   Ueq(max) / Ueq(min) Range        4.4 Ratio
PLAT222_ALERT_3_C NonSolvent Resd 1  H   Uiso(max)/Uiso(min) Range        5.4 Ratio
PLAT911_ALERT_3_C Missing FCF Refl Between Thmin & STh/L=    0.600          2 Report
PLAT918_ALERT_3_C Reflection(s) with I(obs) much Smaller I(calc) .          1 Check
```

Alert level G

```
PLAT002_ALERT_2_G Number of Distance or Angle Restraints on AtSite         20 Note
PLAT003_ALERT_2_G Number of Uiso or Uij Restrained non-H Atoms ...         30 Report
PLAT171_ALERT_4_G The CIF-Embedded .res File Contains EADP Records          3 Report
PLAT172_ALERT_4_G The CIF-Embedded .res File Contains DFIX Records          9 Report
PLAT176_ALERT_4_G The CIF-Embedded .res File Contains SADI Records          2 Report
PLAT178_ALERT_4_G The CIF-Embedded .res File Contains SIMU Records          3 Report
PLAT187_ALERT_4_G The CIF-Embedded .res File Contains RIGU Records          3 Report
PLAT232_ALERT_2_G Hirshfeld Test Diff (M-X)  Ni       --O4A      .        6.3 s.u.
PLAT232_ALERT_2_G Hirshfeld Test Diff (M-X)  Ni       --O3B      .        5.4 s.u.
PLAT301_ALERT_3_G Main Residue  Disorder .............(Resd  1  )         58% Note
PLAT302_ALERT_4_G Anion/Solvent/Minor-Residue Disorder (Resd  2  )       100% Note
PLAT302_ALERT_4_G Anion/Solvent/Minor-Residue Disorder (Resd  3  )       100% Note
PLAT304_ALERT_4_G Non-Integer Number of Atoms in ..... (Resd  2  )       3.55 Check
PLAT304_ALERT_4_G Non-Integer Number of Atoms in ..... (Resd  3  )       2.45 Check
PLAT811_ALERT_5_G No ADDSYM Analysis: Too Many Excluded Atoms ....          ! Info
PLAT860_ALERT_3_G Number of Least-Squares Restraints ............         363 Note
PLAT910_ALERT_3_G Missing # of FCF Reflection(s) Below Theta(Min).          3 Note
PLAT912_ALERT_4_G Missing # of FCF Reflections Above STh/L=  0.600         43 Note
PLAT978_ALERT_2_G Number C-C Bonds with Positive Residual Density.         15 Info
```

0 **ALERT level A** = Most likely a serious problem - resolve or explain
0 **ALERT level B** = A potentially serious problem, consider carefully
4 **ALERT level C** = Check. Ensure it is not caused by an omission or oversight
19 **ALERT level G** = General information/check it is not something unexpected

0 ALERT type 1 CIF construction/syntax error, inconsistent or missing data
6 ALERT type 2 Indicator that the structure model may be wrong or deficient
6 ALERT type 3 Indicator that the structure quality may be low
10 ALERT type 4 Improvement, methodology, query or suggestion
1 ALERT type 5 Informative message, check

It is advisable to attempt to resolve as many as possible of the alerts in all categories. Often the minor alerts point to easily fixed oversights, errors and omissions in your CIF or refinement strategy, so attention to these fine details can be worthwhile. In order to resolve some of the more serious problems it may be necessary to carry out additional measurements or structure refinements. However, the purpose of your study may justify the reported deviations and the more serious of these should normally be commented upon in the discussion or experimental section of a paper or in the "special_details" fields of the CIF. checkCIF was carefully designed to identify outliers and unusual parameters, but every test has its limitations and alerts that are not important in a particular case may appear. Conversely, the absence of alerts does not guarantee there are no aspects of the results needing attention. It is up to the individual to critically assess their own results and, if necessary, seek expert advice.

Publication of your CIF in IUCr journals

A basic structural check has been run on your CIF. These basic checks will be run on all CIFs submitted for publication in IUCr journals (*Acta Crystallographica*, *Journal of Applied Crystallography*, *Journal of Synchrotron Radiation*); however, if you intend to submit to *Acta Crystallographica Section C* or *E* or *IUCrData*, you should make sure that full publication checks are run on the final version of your CIF prior to submission.

Publication of your CIF in other journals

Please refer to the *Notes for Authors* of the relevant journal for any special instructions relating to CIF submission.

PLATON version of 22/12/2019; check.def file version of 13/12/2019

Datablock Ni_mef_1 - ellipsoid plot

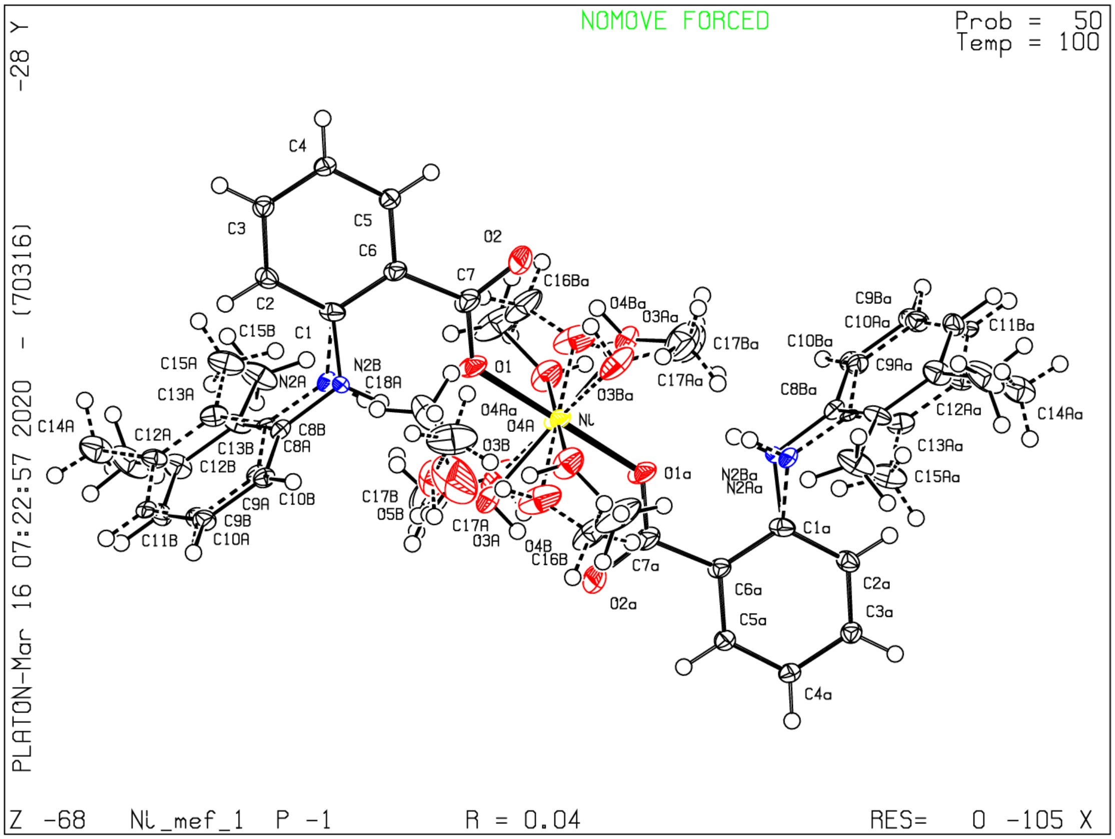

Supplement: Supplementary file 1 [file molecules-25-03099-s001.zip › Structure_4_checkcif.pdf]
